# Validation study of a web-based assessment of functional recovery after radical prostatectomy

**DOI:** 10.1186/1477-7525-8-82

**Published:** 2010-08-05

**Authors:** Andrew J Vickers, Caroline J Savage, Marwan Shouery, James A Eastham, Peter T Scardino, Ethan M Basch

**Affiliations:** 1 Department of Epidemiology and Biostatistics, Health Outcomes Group Memorial Sloan-Kettering Cancer Center, 1275 York Avenue New York, NY 10065 USA; 2 Department of Urology, Memorial Sloan-Kettering Cancer Center, 1275 York Avenue New York, NY 10065 USA

## Abstract

**Background:**

Good clinical care of prostate cancer patients after radical prostatectomy depends on careful assessment of post-operative morbidities, yet physicians do not always judge patient symptoms accurately. Logistical problems associated with using paper questionnaire limit their use in the clinic. We have implemented a web-interface ("STAR") for patient-reported outcomes after radical prostatectomy.

**Methods:**

We analyzed data on the first 9 months of clinical implementation to evaluate the validity of the STAR questionnaire to assess functional outcomes following radical prostatectomy. We assessed response rate, internal consistency within domains, and the association between survey responses and known predictors of sexual and urinary function, including age, time from surgery, nerve sparing status and co-morbidities.

**Results:**

Of 1581 men sent an invitation to complete the instrument online, 1235 responded for a response rate of 78%. Cronbach's alpha was 0.84, 0.86 and 0.97 for bowel, urinary and sexual function respectively. All known predictors of sexual and urinary function were significantly associated with survey responses in the hypothesized direction.

**Conclusions:**

We have found that web-based assessment of functional recovery after radical prostatectomy is practical and feasible. The instrument demonstrated excellent psychometric properties, suggested that validity is maintained when questions are transferred from paper to electronic format and when patients give responses that they know will be seen by their doctor and added to their clinic record. As such, our system allows ready implementation of patient-reported outcomes into routine clinical practice.

## Background

Radical prostatectomy is a mainstay of treatment for early stage prostate cancer. Although associated with excellent rates of cure[1], the procedure leads to erectile and urinary dysfunction. Patients typically experience severe urinary incontinence and erectile dysfunction immediately after surgery, but recover gradually over the course of the first post-operative year[2]. Nonetheless, some patients experience long-term difficulties with sexual function and urinary control[3]. The uncertain nature of return to function is a major source of anxiety for prostate cancer patients recovering from surgery.

There are treatments available for urinary and erectile dysfunction after radical prostatectomy. Pelvic floor exercises ("Kegels") have been shown to improve return of urinary control[4], and procedures such as the male sling can be successful for patients with persistent incontinence[5]. Comparably, PDE5 inhibitors such as Viagra can be used to treat post-prostatectomy erectile dysfunction with some urologists advocating daily use for the first few months after surgery as a form of "penile rehabilitation"[6,7].

Clearly, good clinical care of patients after radical prostatectomy depends on careful assessment of post-operative morbidities. Yet there is accumulating evidence that physicians do not always judge patient symptoms accurately. This has been demonstrated in fields as diverse as chemotherapy[8], primary care[9] and dermatology[10]. Specifically with respect to radical prostatectomy, Sonn et al. compared the results of patient questionnaires with clinical documentation of their urologists in 1,366 men following curative treatment for prostate cancer, about 70% of whom underwent surgery. Physicians consistently underestimated patient dysfunction when compared to questionnaire responses from patients. For example, at long term follow-up, 42% of patients reported urinary dysfunction whereas physicians documented urinary dysfunction only in about half as many patients (22%). Similarly, whereas nearly all patients (94%) reported some level of erectile dysfunction, only 62% of patients were coded as such by their urologist[11].

While patient-reported outcomes are clearly preferable, considerable logistical problems are associated with their integration into routine clinical care. Paper questionnaires need to be administered to patients, checked and then the responses tallied, for example, by summing specific questions to calculate domain specific scores, with the results then entered into the clinical record. All this must be done by busy clinic staff in a timely manner such that the doctor can access the questionnaire results before the consultation.

At Memorial Sloan-Kettering Cancer Center, we have started to integrate electronic recording of patient-reported questionnaires into our clinics. These allow automatic checking of patient responses and direct porting of summary information into the clinic record. Known as the STAR system ("Symptom Tracking and Reporting"), this system was initially developed for use by patients receiving outpatient chemotherapy and was found to be feasible for both clinic-based and home-based internet reporting in diverse populations, including those with no prior computer experience, lower educational levels, and high symptom burdens[12,13]. Use of the platform was expanded into the clinical trial setting with ongoing national multi-center evaluations in the National Cancer institute-sponsored cooperative groups, and a system in development for the NCI under contract based on the STAR model, called the PRO-CTCAE[14].

STAR is a flexible interface which allows administration of questions to patients via the web, either from clinic-based computers or from home[15]. Reminder emails can be triggered to patients who do not complete scheduled questionnaires. The platform sends automated notifications to study staff if a patient misses a scheduled questionnaire, or reports a concerning symptom which merits clinical evaluation. Data collected by STAR are exported to the electronic health record and clinical trials database for clinicians and investigators to view results.

We adapted the STAR system to record data on functional recovery after radical prostatectomy. Here we report our initial experience of obtaining patient-reported outcomes through a web interface. In particular, we were interested in patient response rates and in evaluating the psychometric validity of our instrument.

## Methods

### Development of the instrument

We have previously validated a questionnaire for post-treatment recovery in prostate cancer patients[16]. This questionnaire was deemed too long and complex for web implementation as it included 68 questions, some of which had up to 18 response options. Accordingly, we choose a subset of questions for our web-instrument. We chose six questions from the erectile function domain that constitute the International Index of Erectile Function-6 (IIEF-6)[17], a well-validated and widely used instrument. We then analyzed data from questionnaire responses of radical prostatectomy patients to identify 6 of 16 questions from the urinary domain that demonstrated good internal consistency (Cronbach's alpha of 0.82) and, when summed, had excellent correlation with the full urinary domain score (0.90). Bowel symptoms are rare after radical prostatectomy, and we found that just two questions about bother from bowel symptoms that had good correlation (0.91) with the total of all questions from the bowel domain score. We added a simple 0 - 10 scale of global health related quality of life to give a total of 15 questions.

Having chosen the items for our instrument, we added two interactive features. First, the IIEF6 asks several questions about erectile function that depend on sexual activity. For example, a question about erection frequency and another about erection hardness both have "no sexual activity" as a response. Accordingly, we designed the questionnaire so that if a patient responded "no sexual activity" to the first question about erections, further questions were skipped and scored as zero. Second, assessment of recovery after radical prostatectomy is subject to what is known as interval censoring. A patient given a questionnaire at three and six months and who, for example, reports use of incontinence pads at the first but not the second questionnaire, is typically recorded as having regained continence at six months. In truth, the patient likely stopped using pads at some point in the preceding three month period, and not on the exact day that the questionnaire was administered. Thus, when a patient first responds that he is not using pads, an additional question is implemented concerning when the patient first stopped needing pads (within the past month; 1 - 2 months; 2 - 3 months; more than 3 months). Similarly, when a patient first reports erections sufficient for penetration, he is asked about when this was first achieved. Additional file 1 shows the instrument and scoring.

### Informatics implementation

STAR hardware includes two servers that reside in the MSKCC New Jersey Data Center. One is the web server and is located in the "DMZ" where it is accessible to the Internet. The other server is the database server and is completely protected by the MSKCC firewall.

The web server uses Microsoft Internet Information Services (IIS) and Secure Sockets Layer (SSL) encryption to protect the data through the Internet. The web application is developed using Microsoft .Net technologies and uses the highest security and privacy features that exist to date.

The database server hosts Microsoft SQL server where the data is stored and backed up daily. All information in STAR is saved in the database where it is completely protected by the firewall.

STAR database shares information with CAISIS, an open source, web-based cancer data management system that is used for clinical management of patients at MSKCC. Patient information is entered in CAISIS and "pulled" by STAR. STAR in turn registers the patients automatically and emails them in due time to take the survey, based on surgery and appointment dates provided by CAISIS. Once the survey is taken, STAR will "push" the data back to CAISIS where it can be seen by clinicians and downloaded for research purposes.

### Implementation of the web-based tool in clinical practice

Patients who provide an email address to the hospital are sent an email inviting them to complete a questionnaire at 3, 6, 9, 12, 18, 24, 36, and 48 months after surgery. However, patients with a clinic appointment up to six weeks before any of these time points are sent reminder emails two weeks prior to their appointment so that their responses are available to their urologist in good time. The text of the email reminder is given in additional file 2: the key point is that patients are told that their answers will go directly into their clinic record so that their doctors can see how they are doing.

As an attempt to improve clinical practice, the web-based tool was not subject to an IRB protocol. Data for this evaluation was obtained under an IRB waiver for routinely collected data to be used for research purposes.

### Statistical Considerations

Our initial aim was to investigate the validity of the STAR questionnaire to assess functional outcomes following radical prostatectomy. For assessment of internal consistency within domains, Cronbach's alpha coefficient was calculated for all domains of the questionnaire, except overall quality of life, which is a single-item measure.

To assess instrument validity, we evaluated the association between survey responses and known predictors of sexual and urinary function. To account for men who completed more than one survey, we used multivariable generalized estimating equations. Pre-specified predictors included time from surgery, age at surgery, nerve sparing status (none, unilateral or bilateral), comorbidities (0, 1 or >1). Separate models were built for the outcomes of urinary and sexual function. For these analyses, sexual and urinary function domains were rescaled to a 0 - 100 range, in order to allow direct comparison. To account for a possible non-linear relationship between recovery of function and either time from surgery or age, we included non-linear terms (restricted cubic splines with knots at the tertiles). We hypothesized that valid measures of erectile and urinary function would show decreasing scores with age and comorbidity, and increasing scores with time from surgery, and nerve sparing surgery.

We also compared the surgeon and patient-reported assessments of function. We restricted this analysis to occasions when the patient- and physician-reported assessment where within six weeks of one another. We used the first eligible assessment time for patients with more than one time point for which both patient- and physician reported data were available. Physician-reported erectile and urinary function are on a five point scale. Sexual function was defined as physician-assessed score of 1 or 2 (normal, full erections or full, but diminished erections satisfactory for sexual activity); continence as classified as physician-assessment of 1 (no pads). All analyses were conducted using Stata 11.0 (Stata Corp., College Station, Texas).

## Results

The STAR system was implemented at MSKCC in one surgeon's clinic as a pilot in April 2009. The system was made available in all clinics in June 2009. All new patients undergoing radical prostatectomy with Email addresses were eligible as well as patients treated up to four years before the start of the project (April 2005). The database was closed for analysis in February 2010. A total of 1,581 men had been sent at least one email inviting them to complete an online questionnaire in this time (approximately 50% of patients provided an email address). Of these, 1,235 completed at least one survey for an overall response rate of 78%. Russian and Spanish language versions of the instrument are available by clicking a link on the STAR portal; however, fewer than 1% of users accessed the questionnaire in a foreign language. Table 1 shows the characteristics of the men who did and did not complete a survey. Overall there were no obvious differences between groups (Table 1).

**Table 1:** Summary of patient characteristics.

	All men who completed a questionnaire N = 1235	Men who were invited, but did not complete questionnaire N = 346
Age at surgery (years)	62 (57, 67)	60 (56, 65)

PSA (ng/ml)	5.00 (3.48, 6.95)	5.30 (3.98, 7.14)

Pathologic Gleason Score		

<= 6	250 (20%)	34 (24%)

7	816 (66%)	90 (63%)

>= 8	169 (14%)	19 (13%)

Lymph node involvement		

LN+	91 (8%)	9 (6%)

LN-	1019 (83%)	119 (83%)

No LND	6 (1%)	0 (0%)

*Unknown/missing*	125 (10%)	15 (10%)

Extracapsular extension	659 (58%)	73 (55%)

Positive Surgical Margins	153 (12%)	13 (9%)

Seminal Vesicle Invasion	85 (7%)	7 (5%)

Nerve sparing status		

None	235 (19%)	24 (17%)

Unilateral	174 (14%)	24 (17%)

Bilateral	826 (67%)	95 (66%)

Number of comorbidities		

0	475 (38%)	46 (32%)

1	421 (34%)	58 (41%)

2	249 (20%)	28 (20%)

3+	89 (7%)	11 (8%)

All values are median (IQR) or frequency (proportion).

Nearly all surveys were completed in their entirety (n = 1761; 93%); 78 (4%) questionnaires included one missed question and only 48 (3%) included more than one missed question.

Table 2 shows Cronbach's alpha coefficient 0.84, 0.86 and 0.97 for bowel, urinary and sexual function respectively. To test whether the three questionnaires were measuring separate aspects of function, we calculated correlations for all pairs of questions. Survey questions assessing the same function (potency, continence or bowel function) were more highly correlated (mean within-function correlation coefficients of 0.83, 0.54 and 0.74 for erectile, urinary and bowel function) than measures assessing different functions (mean between-function correlation coefficients of 0.17, 0.15 and 0.31 for erectile and urinary, erectile and bowel, and urinary and bowel respectively).

**Table 2:** Summary of response rate and average scores for each domain.

	Number of items	Median (IQR) score (original scaling)	Range of scores (original scaling)	Cronbach's alpha
Sexual function	6	8 (3, 23)	1-30	0.97

Urinary function	5	18 (15, 20)	0-21	0.86

Bowel function	2	8 (7, 8)	0-8	0.84

Overall quality of life	1	8 (7, 9)	0-10	-

Men who completed more than one survey are represented more than once.

We hypothesized that, if our instrument was valid, there should be a positive correlation between function and quality of life, with urinary function having a higher correlation than sexual function. This is indeed what we found: the correlations between total sexual and urinary function and overall quality of life were 0.27 and 0.47, respectively.

Table 3 shows that age, time for surgery, and nerve sparing status were all significantly associated with both urinary and sexual function in the hypothesized direction. An increasing number of comorbidities was significantly associated with poorer urinary and sexual function (p = 0.028 and p = 0.019, respectively). Men with three or more comorbidities had on average a 7 point lower urinary score and a 13 point lower sexual function score than those without any comorbidity.

**Table 3:** Predictors for urinary and sexual function scores.

	Urinary function		Sexual function	
	**Coefficient (95% CI)**	**P value**	**Coefficient (95% CI)**	**P value**

Age	*	0.0011	*	<0.001

Time from surgery	*	<0.001	*	<0.001

Nerve sparing status		<0.001		<0.001

None	Reference		Reference	

Unilateral	9.0 (3.8, 14.2)		3.0 (-4.8, 10.7)	

Bilateral	9.1 (5.0, 13.1)		17.2 (11.2, 23.2)	

Comorbidities		0.028		0.019

0	Reference		Reference	

1	2.0 (-1.4, 5.3)		-3.3 (-8.3, 1.7)	

2	-1.4 (-5.4, 2.7)		-6.5 (-12.5, -0.4)	

3+	-6.9 (-13.0, -0.8)		-13.1 (-22.2, -3.9)	

Scores have been rescaled so that the maximum scores for both urinary and sexual function are 100.

**Figure 1:**
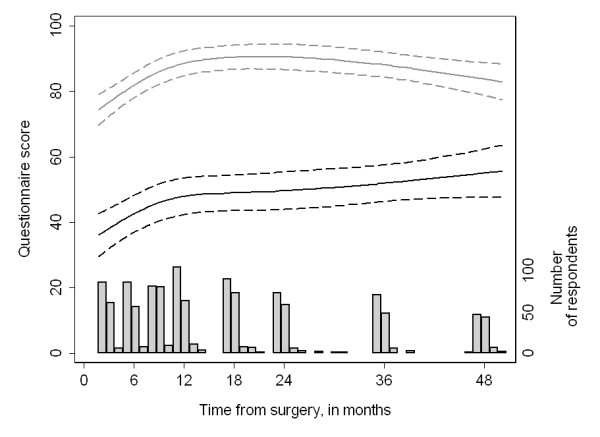
**Recovery of sexual (black lines) and urinary (gray lines) function by time from surgery**. Values are reported for a man with one comorbidity who received bilateral nerve sparing and was age 62 at surgery. Scores have been rescaled so that the maximum scores for both urinary and sexual function are 100. Dashed lines are 95% CI.

**Figure 2:**
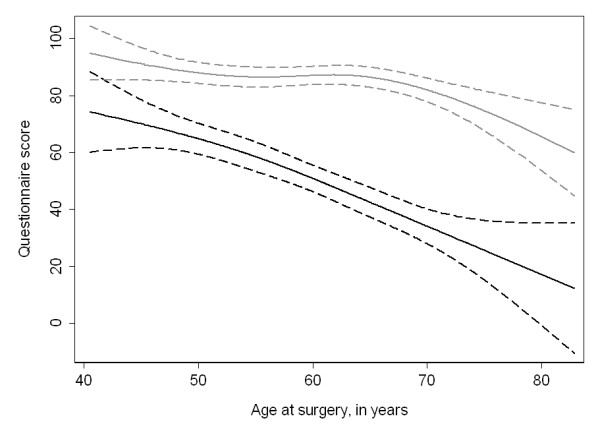
**Recovery of sexual (black lines) and urinary (gray lines) function by age at the time of surgery**. Values are reported for a man 12 months after surgery who had one comorbidity and who received bilateral nerve sparing. Scores have been rescaled so that the maximum scores for both urinary and sexual function are 100. Dashed lines are 95% CI.

*includes non-linear terms (see figure 1 and 2)

Figure 1 illustrates the association between time from surgery and recovery of function. We hypothesized that erectile and urinary function would increase with time from surgery, and that erectile function would continue to improve for a longer time than urinary function. Both erectile and urinary function improve significantly (both p < 0.001), particularly within the first year after surgery; as hypothesized, erectile function continues to improve beyond one year, whereas urinary function plateaus.

Our second hypothesis was that both erectile and urinary function would decrease with age, and that this decrease would be larger for sexual as compared to urinary function. Figure 2 illustrates older men have much lower sexual function one year after surgery: at age 55 the mean adjusted 12 month erectile function score was 58 (95% CI: 53, 64); by age 70 it had declined to 34 (95% CI: 28, 40). Urinary function appeared to remain relatively constant until about age 70 (adjusted urinary scores were 86 [95% CI: 83, 90] and 82 [95% CI: 78, 86] at age 55 and 70, respectively), after which point we saw small decreases in reported function (adjusted score at age 75 was 75 [95% CI: 68, 82]).

Our third hypothesis was that patient-reported sexual and urinary function scores would predict physician-reported assessments of potency and continence. Among those with patient and physician reported scores that were measured within six weeks of each other (n = 365 and 469 for sexual and urinary function, respectively), we found that patient-reported sexual and urinary function scores predicted physician-assessed function with high discrimination (sexual function AUC: 0.86 and urinary function AUC: 0.87; p < 0.001 for both).

## Discussion

We have found that web-based assessment of functional recovery after radical prostatectomy is practical and feasible. The tool clearly has good patient acceptability, as evidenced by the very high response rate of 78%. Indeed, we believe that this is an underestimate as it includes as non-responders patients who gave non-functioning email addresses. For example, we became aware that some of the addresses given by patients were those of relatives.

The patient-reported outcomes instrument has excellent psychometric properties. There was very high internal consistency within domains, and excellent validity: domain scores not only predicted overall health related quality of life, but do so exactly as hypothesized, with urinary function showing a stronger relationship than sexual function; there was a tight association between domain scores and physician assessments; correlation between items within domains was much higher than correlation between items from different domains; domain scores discriminated between groups known to differ, such as decreases in erectile function with age and nerve resection and increases in urinary function with time from surgery. These findings might not be surprising given that our instrument consists of a subset of questions from a previously validated questionnaire. Nonetheless, we have shown that validity is maintained when the questions are transferred from paper to electronic format. Moreover, our findings show that validity is not strongly affected when patients give responses that they know will be seen by their doctor and added to their clinic record, in contrast to providing confidential answers, as is typical in research studies.

Our system was costly to establish, requiring considerable programming time, but incurs near zero marginal costs for new patients. A member of the clinic staff has been assigned to ensure that the salutation field is appropriate (e.g. "Dear Mr. Jones" rather than "Dear Mr. Jones Jr.") and to be the first point of call for patient enquiries. Some of these enquiries are technical and so are forwarded to a programmer. But the total number of enquiries is low, approximately 1 - 2 per month, and few enquires require more than a couple of minutes to resolve.

An obvious limitation of our system is that it is available only for individuals with email access. Accordingly, we are currently developing a version that can be used by patients in clinic. We will purchase inexpensive notebook computers that we will then "lock down" so that only our web-interface is available. Patients will be logged in by clinic staff, who will be available to advise patients if they have difficulties. Our previous experience of implementing STAR in chemotherapy clinics at MSKCC is that patients find it no more challenging to use than an ATM machine[15]. Currently, an automated telephone system (interactive voice response system) is being developed as an optional add-on to STAR to administer items by telephone.

We have not discussed assessment of baseline function in this paper as our focus has been the evaluation of post-treatment questionnaires. Assessment of function before surgery is naturally a key part of good clinical care, and essential for interpreting a patient's post-operative function. Baseline data are currently collected using a paper version of the web-based questionnaire. Paper forms are used as we are yet to establish systems for email contact before a patient's first session. We will transition to the web-based system this year when computers are made available in the clinic, as discussed above.

Our focus on the psychometric evaluation of the web-based questionnaires has also left unaddressed the issue of how the system is used by surgeons, and the degree to which they find it useful. We plan to evaluate systematically how surgeons use the system to guide patient care and follow up.

## Conclusions

We have developed a web-based assessment of functional recovery after radical prostatectomy. We have obtained very high response rates, and the instrument demonstrates excellent psychometric validity. As such, our system allows ready implementation of patient-reported outcomes into routine clinical practice.

## Competing interests

The authors declare that they have no competing interests.

## Authors' contributions

AV, PS, JE and EB were involved in the conception and design of the study. MS created the online database program and collected the data for analysis. AV and CS performed the statistical analyses and drafted the manuscript. All authors read and approved the final manuscript.

## Supplementary Material

Additional file 1 **Questionnaire**. List of questions used in web-based questionnaire. Click here for file

Additional file 2 **Email to patient**. An example of an email sent to patients providing them with information about the questionnaire. Click here for file
