# Supplementary material for: Validation study of a web-based assessment of functional recovery after radical prostatectomy
Source: Health Qual Life Outcomes. 2010 Aug 5;8:82. doi: 10.1186/1477-7525-8-82 (PMC2927505; doi:10.1186/1477-7525-8-82)
Supplement: Additional file 1 — Questionnaire. List of questions used in web-based questionnaire. [file 1477-7525-8-82-S1.DOCX]

Appendix 1

| These questions are about your sexual function. This includes foreplay, masturbation, intercourse with partner, and other forms of sexual activity during the last four (4) weeks. |  |
| --- | --- |
|  |  |
| During the last four (4) weeks, how often did you take a pill like Viagra? |  |
| Never |  |
| Sometimes |  |
| Regularly |  |
| Please answer the following questions regardless if you use pills such as Viagra. If you are currently taking pills such as Viagra, answer the following questions based on your experiences with the medication. |  |
|  |  |
| During the last four (4) weeks, rate your confidence that you could get and keep an erection. |  |
| Very Poor |  |
| Poor |  |
| Fair |  |
| Good |  |
| Very Good |  |
|  |  |
| During the last four (4) weeks, how often were you able to get an erection during sexual activity? |  |
| No sexual activity |  |
| Almost never/never |  |
| A few times (less than half the time) |  |
| Sometimes (about half the time) |  |
| Most times (more than half the time) |  |
| Almost always/always |  |
| Over the last four (4) weeks, when you had erections with sexual stimulation, how often were your erections hard enough for penetration (entering your partner)? |  |
| No sexual activity |  |
| Almost never/never |  |
| A few times (less than half the time) |  |
| Sometimes (about half the time) |  |
| Most times (more than half the time) |  |
| Almost always/always |  |
| Over the last four (4) weeks, when you attempted sexual intercourse, how often were you able to penetrate (enter) your partner? |  |
| I did not attempt intercourse |  |
| Almost never/never |  |
| A few times (less than half the time) |  |
| Sometimes (about half the time) |  |
| Most times (more than half the time) |  |
| Almost always/always |  |
| Over the last four (4) weeks, during sexual intercourse, how often were you able to maintain your erection after you had penetrated (entered) your partner? |  |
| Did not attempt intercourse |  |
| Almost never/never |  |
| A few times (less than half the time) |  |
| Sometimes (about half the time) |  |
| Most times (more than half the time) |  |
| Almost always/always |  |
| Over the last four (4) weeks, during sexual intercourse, how difficult was it to maintain your erection to complete intercourse? |  |
| Did not attempt intercourse |  |
| Extremely difficult |  |
| Very difficult |  |
| Difficult |  |
| Slightly difficult |  |
| Not difficult |  |
| These questions are about your urinary function. |  |
|  |  |
| How many pads or adult diapers per 24-hour period did you use to control urine leakage during the last four (4) weeks? |  |
| None (or no leakage) |  |
| An occasional pad or protective material |  |
| 1 pad per 24-hour period |  |
| 2 pads per 24-hour period |  |
| 3 or more pads per 24-hour period |  |
| Adult diaper(s) |  |
| In the past four (4) weeks, have you dripped or leaked urine when you had the urge to urinate, before you could get to the bathroom? |  |
| Rarely or not at all |  |
| About once a week |  |
| More than once a week |  |
| About once a day |  |
| More than once a day |  |
| In the past four (4) weeks, when you have had the feeling you need to urinate, how often have you found it difficult to postpone urination? |  |
| None of the time or rarely |  |
| Less than half the time |  |
| About half the time |  |
| More than half the time |  |
| Almost always |  |
| In the past four (4) weeks, how often have you had to urinate again less than two hours after you finished urinating? |  |
| None of the time or rarely |  |
| Less than half of the time |  |
| About half of the time |  |
| More than half of the time |  |
| Almost always |  |
| Overall, how big a problem has your urinary function been for you during the last four (4) weeks? |  |
| No problem |  |
| Very small problem |  |
| Small problem |  |
| Moderate problem |  |
| Big problem |  |
| These questions are about your bowel function during the last four weeks. | |
|  |  |
| Overall, how big a problem have your bowel habits been for you during the last four (4) weeks? |  |
| No problem |  |
| Very small problem |  |
| Small problem |  |
| Moderate problem |  |
| Big problem |  |
| Over the past four (4) weeks, how often have bowel problems or bowel pain made it difficult to enjoy your life? |  |
| Never |  |
| Rarely |  |
| Some of the time |  |
| Frequently |  |
| Most of the time |  |
| General health related quality of life |  |
|  |  |
| Using the scale below, select the number that best indicates how you feel about your current state of health. |  |
| 0 - 10 |  |
| *The following three questions are included depending on the patient’s responses to the above questions.*  You have responded that you have not had any sexual activity in the last four weeks. This includes foreplay, masturbation, intercourse with partner, and other forms of sexual activity. If you have not had any sexual activity at all in the past four weeks, press Next to proceed to questions about urinary function. Otherwise, press Previous. |  |
|  |  |
| When did you stop needing pads to control urinary leakage? |  |
| Within the last month |  |
| Between 1 and 2 months ago |  |
| Between 2 and 3 months ago |  |
| More than three months ago |  |
| When did you first achieve an erection sufficient for penetration during intercourse? |  |
| Within the last month |  |
| Between 1 and 2 months ago |  |
| Between 2 and 3 months ago |  |
| More than three months ago |  |
