# Supplementary material for: Validation study of a web-based assessment of functional recovery after radical prostatectomy
Source: Health Qual Life Outcomes. 2010 Aug 5;8:82. doi: 10.1186/1477-7525-8-82 (PMC2927505; doi:10.1186/1477-7525-8-82)
Supplement: Additional file 2 — Email to patient. An example of an email sent to patients providing them with information about the questionnaire. [file 1477-7525-8-82-S2.DOCX]

Dear Mr. PATIENT NAME HERE.

I certainly hope that this letter finds you well. I am interested in hearing about how you are doing. I would be very grateful if you could complete the following questionnaire. To start please [click here](https://webcore.mskcc.org/) (your user name is your email address and your password is “MSKStar123”) and it will take you to a series of questions about your health.

This information will be automatically entered in our clinical database. I assure you that your information will be kept confidential in accordance with the Health Insurance Portability and Accountability Act (HIPAA) of 1996.

Please be aware that I may not review the information you entered until your upcoming visit. Your responses are stored in the clinical database and will be discussed with you at your next clinic appointment. Should you have any questions about your health or would like to review any of your responses sooner, please telephone my office.

Thank you for your kind cooperation. I believe that your participation will benefit you by helping me to see how you are doing.

Please note that if you select “reply” to these email, your message will go to an unattended Email box. If you have any questions or concerns, please telephone my office.

Sincerely,

DOCTOR NAME HERE MD

Memorial Sloan-Kettering Cancer Center

Note: If you do not wish to receive any further emails from me, you can unsubscribe by [clicking here](https://webcore.mskcc.org/Subscribe.aspx).
